# Advancements in Cold Spray Additive Manufacturing: Process, Materials, Optimization, Applications, and Challenges

**DOI:** 10.3390/ma17225431

**Published:** 2024-11-07

**Authors:** Abishek Kafle, Raman Silwal, Bikram Koirala, Weihang Zhu

**Affiliations:** 1 Department of Mechanical and Aerospace Engineering, University of Houston, Houston, TX 77204, USA; akafle@cougarnet.uh.edu (A.K.); rsilwal@cougarnet.uh.edu (R.S.); bkoirala@cougarnet.uh.edu (B.K.); 2 Department of Engineering Technology, University of Houston, Houston, TX 77204, USA; 3 Department of Industrial Engineering, University of Houston, Houston, TX 77204, USA; 4 Department of Electrical and Computer Engineering, University of Houston, Houston, TX 77204, USA

**Keywords:** CSAM, additive manufacturing, cold spray, high-strength manufacturing, machine learning

## Abstract

Cold spray additive manufacturing (CSAM) is a cutting-edge high-speed additive manufacturing process enabling the production of high-strength components without relying on traditional high-temperature methods. Unlike other techniques, CSAM produces oxide-free deposits and preserves the feedstock’s original characteristics without adversely affecting the substrate. This makes it ideal for industries requiring materials that maintain structural integrity. This paper explores strategies for improving material quality, focusing on nozzle design, particle size distribution, and fine-tuning of process parameters such as gas pressure, temperature, and spray distance. These factors are key to achieving efficient deposition and optimal bonding, which enhance the mechanical properties of the final products. Challenges in CSAM, including porosity control and achieving uniform coating thickness, are discussed, with solutions offered through the advancements in machine learning (ML). ML algorithms analyze extensive data to predict optimal process parameters, allowing for more precise control, reduced trial-and-error, and improved material usage. Advances in material strength, such as enhanced tensile strength and corrosion resistance, are also highlighted, making CSAM applicable to sectors like aerospace, defense, and automotive. The ability to produce high-performance, durable components positions CSAM as a promising additive-manufacturing technology. By addressing these innovations, this study offers insights into optimizing CSAM processes, guiding future research and industrial applications toward more efficient and high-performing manufacturing systems.

## 1. Introduction

Cold-spray (CS) technology is a solid-state material deposition process increasingly used for coatings and, recently, additive manufacturing due to its ability to preserve the original properties of materials by avoiding high-temperature processes [1,2,3,4]. Unlike conventional methods, CSAM leverages the high-velocity impact of solid-state particles to build or repair components, thereby preserving the intrinsic properties of the materials. This innovative process has been increasingly adopted across various industries, including aerospace, automotive, and biomedical, due to its ability to produce dense, high-quality deposits with enhanced mechanical properties [5]. Since there is no burning and no greenhouse gas emissions, carbon sequestration is considered environmentally friendly [6]. Since CS does not melt, any remaining stress from shrinkage upon solidification is eliminated. The integration of advanced technologies, such as machine learning for process optimization, further amplifies the potential of CSAM, making it a viable alternative to traditional additive-manufacturing techniques. We refer to these excellent review articles to obtain a comprehensive insight into the CSAM technology: [1,7,8,9,10]. This review aims to provide a comprehensive overview of recent advancements in CSAM, focusing on process optimization, material selection, and the application of machine learning to enhance the efficiency and reliability of the manufacturing process.

The rest of this review paper is organized as follows: Section 2 explains cold spray technology. Section 3 describes how cold spray technology is used for additive manufacturing. Section 4 explains the materials for CSAM. Section 5 discusses process optimization, in particular, high-strength manufacturing, and the integration of machine-learning techniques to enhance these processes. Section 6 explores the applications. Section 7 brings up the challenges. Section 8 draws the conclusions.

Cold spraying (CS) involves accelerating powder particles through a high-velocity gas stream, typically nitrogen or helium, which impacts a substrate and forms a dense, solid-state layer [6]. Although the process gas is heated to provide higher acceleration and to facilitate particle deformation through thermal softening, the feedstock remains in a solid state throughout the entire process, which is why it is termed ‘cold’ spraying. The primary advantage of cold spraying (CS) is that it mitigates the issues linked with high-temperature material processing, such as oxidation and undesirable structural changes. Cold spraying (CS) differs significantly from laser, welding, and other thermal spray processes, as it does not alter the properties of the feedstock powder by heating or melting. Instead, the powder is kept below its recrystallization temperature throughout the spraying process. A fundamental difference exists between conventional thermal spray techniques and the cold spray process. Thermal spray techniques rely on both thermal and kinetic energies for coating formation, whereas cold spray utilizes only kinetic energy. The cold spray technique is particularly suitable for depositing materials with smaller heat capacities, such as copper, aluminum, and titanium, which are softer and sensitive to oxygen [7]. This process effectively minimizes issues like oxidation, coating porosity, phase transformations, formation of heat-affected zones (HAZs), and thermal residual stresses that are common in other thermal-spray-coating processes. Additionally, it enables the deposition of highly dense and thick coatings [8].

**Figure 1:**
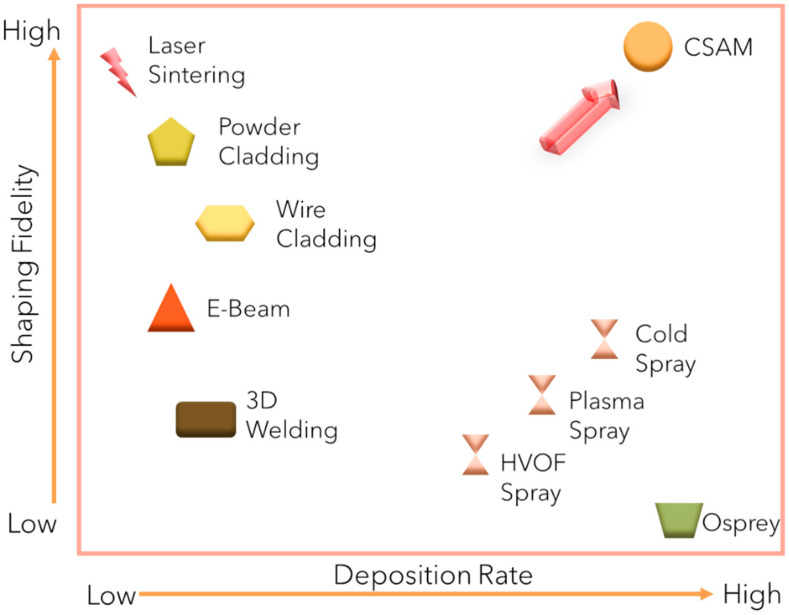
Comparison of shaping fidelity of different fusion-based manufacturing processes.

The cold spray process is utilized not only for coating deposition but also for the repair of critical components [9]. It is increasingly recognized as an alternative solution for repairing complex parts, particularly in the aeronautical, defense, and turbine industries [10]. In the aerospace sector, the use of cold spray for repairing Seahawk helicopter modules has achieved cost reductions of up to 50 percent. The process proved effective for multiple modules and followed a certified protocol, encompassing various rigorous tests, including those for corrosion resistance, tensile strength, compression/bearing capacity, shear strength, fatigue resistance, residual stress, impact durability, and hydrogen embrittlement. Cold spray technology is considered optimal for repairing large surfaces with complex shapes requiring precise deposition for thin layer repair and uniform coating. Repair of turbine blades is another prominent application of cold spray technology [11]. Key process parameters influencing the CSAM process include gas pressure and temperature, powder feed rate, nozzle design, and standoff distance. These parameters must be carefully optimized to achieve desired material properties and deposition efficiency. Figure 1 provides a general overview of shaping fidelity of different fusion-based manufacturing processes. For CSAM, the deposition rate is higher, and the shaping fidelity is higher. 

## 2. Mechanisms and Key Parameters in Cold Spray Additive Manufacturing

Cold spray has been successfully advanced as an additive-manufacturing technique, allowing for producing free-standing metal components and the repair of damaged ones. This progress offers a fresh perspective on conventional additive-manufacturing methods and considerably expands the applications of cold spray technology. As a novel addition to the additive-manufacturing landscape, CSAM inherits all the advantages of traditional cold spray technology. CSAM processes have unique advantages and strengths in comparison to other additive manufacturing processes. 

**Figure 2:**
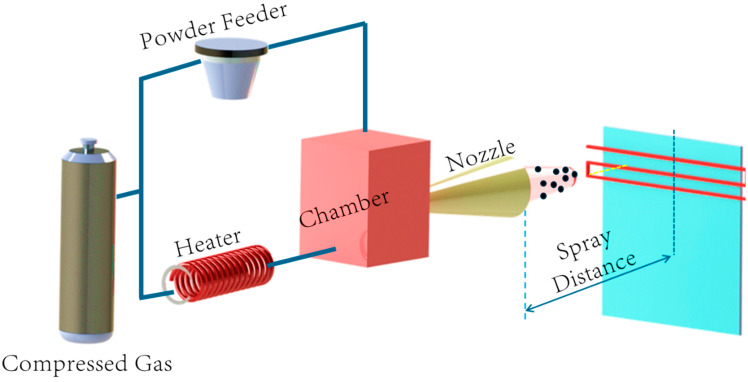
Cold spray additive manufacturing.

Figure 2 describes the working principle of CSAM. A few commercially available CSAM systems are the Gen IIITM Portable High-Pressure Cold Spray System from VRC Metal Systems (Box Elder, SD, USA), LightSPEE3D and WarpSPEE3D from SPEE3D (Melbourne, Victoria, Australia), and Titomic Kinetic Fusion (TKF1000, TKF9000, TKF2200) (Macomb Township, MI, USA). Titomic’s systems have a large build volume of 1 m × 1 m and use a high-velocity cold spray method to deposit metal quickly and with great strength, making them appropriate for a variety of metals and composites. With its unique cold spray technology, SPEE3D printers deposit metal up to 100 times faster than conventional methods, and they provide a build space of 1 m × 1 m × 0.7 m with WarpSPEE3D. The Gen IIITM solution from VRC Metal Systems is a small and lightweight option that measures 182.9 cm × 81.3 cm × 167.6 cm. It is perfect for on-site repairs and uses high-pressure depositions for direct coating and restoration without the need for disassembly.

### 2.1. Mechanism of Deposit Formation

CSAM involves a unique deposit-formation process characterized by the high-velocity impact of powder particles onto a substrate, creating a solid layer without melting the materials. The process begins by introducing powder particles into a high-pressure gas stream, which is directed through a de Laval nozzle to reach supersonic velocities. These high-velocity particles strike the substrate and undergo plastic deformation, creating localized high-pressure zones that facilitate metallurgical bonding without melting. The bonding is enhanced by the removal of surface oxides and contaminants due to the high impact forces, resulting in clean surfaces that promote strong adhesion through mechanical interlocking, adiabatic shear instability, and localized recrystallization. This layer-by-layer deposition approach allows for precise control of deposit thickness and geometry, producing dense, high-strength deposits with fine-grained microstructures. 

The cold spray process relies on high-velocity particles impacting the substrate, generating significant plastic deformation at the point of contact. Despite the process occurring well below the melting temperature of the materials involved, the localized temperatures at the points of impact may rise significantly due to the high kinetic energy of the particles. This localized heating is primarily driven by adiabatic shear, friction, and deformation, all of which result in the dissipation of mechanical energy as heat. The result is an extremely short-lived temperature rise that remains confined to microscopic regions at the interface between the particles and the substrate.

This phenomenon of localized heating is critical to the cold spray process, as it plays a pivotal role in enabling solid-state bonding without the material reaching its melting point. The localized shear forces induce severe plastic deformation, and combined with frictional heating, they promote mechanical interlocking and atomic diffusion across the impacted surfaces. These processes contribute to the formation of metallurgical bonds at the interface, allowing for the deposition of particles without compromising the structural integrity of the substrate or causing undesirable phase changes. It is important to note that while temperature spikes may occur at the impact site, they are highly localized and transient. These spikes typically dissipate within nanoseconds, ensuring that the overall temperature of the component or the surrounding atmosphere remains largely unaffected. This localized nature of the heating ensures that cold spray remains a solid-state process, preserving the material’s original properties, such as phase composition, grain structure, and mechanical strength, which are often altered in conventional thermal spray methods.

Recent studies have delved into the role of high strain rates and their contribution to the heat generation and bonding mechanism in cold spray. These studies reveal that in extreme cases, the instantaneous temperature at the impact zone can approach significant fractions of the melting temperature, though not long enough to induce melting. This balance between heat generation and rapid dissipation underlines the importance of understanding the thermomechanical behavior of particles during deposition. Moreover, advancements in high-fidelity computational modeling, such as finite element analysis (FEA) and molecular dynamics simulations, have provided deeper insights into the transient thermal phenomena occurring during cold spray. These models have demonstrated how parameters such as particle velocity, size, material properties, and substrate characteristics influence the temperature distribution and bonding efficiency at the micro-scale. Such simulations allow for the prediction and optimization of particle–substrate interactions, reducing the need for extensive experimental trials.

In practice, the control of impact velocity and particle morphology has been found to be key in minimizing any adverse effects of localized heating. Excessively high-impact velocities may lead to particle erosion or fracture, while velocities too low may prevent proper bonding. Thus, fine-tuning these parameters is crucial to maximizing deposition efficiency while maintaining the integrity of the deposited layer. Additionally, the cold-spray process’s ability to maintain low operational temperatures offers significant advantages over traditional thermal spray techniques, particularly for heat-sensitive materials such as aluminum alloys, composites, and polymers. By preventing the bulk heating of the substrate, cold spray enables the deposition of these materials without degrading their mechanical properties or introducing residual thermal stresses. This makes cold spray particularly useful in applications such as the repair of aerospace components, coatings for corrosion and wear resistance, and the fabrication of complex additive-manufactured structures.

**Figure 3:**
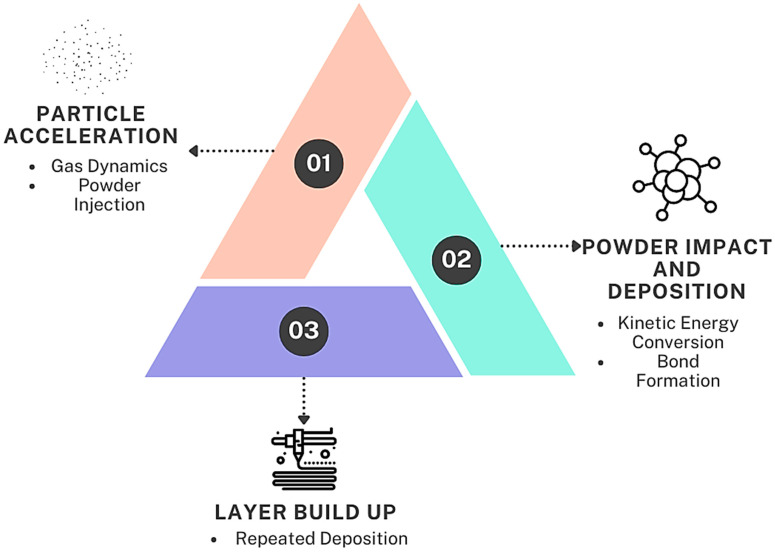
Mechanism of deposit formation.

The absence of melting minimizes thermal stress and oxidation, ensuring high-purity and high-integrity deposits. This process is suitable for repairing components, creating protective coatings, and fabricating complex geometries, with the versatility to use various materials, including metals, composites, and ceramics, making it ideal for temperature-sensitive applications and achieving high-performance coatings and structures. The core mechanism of CSAM is the high-velocity impact of particles onto a substrate. The process is described in Figure 3.

High-pressure gas, such as nitrogen or helium, is heated and accelerated through a converging–diverging nozzle (de Laval nozzle). The choice of gas affects the achievable particle velocity, with helium generally providing higher velocities due to its lower molecular weight. Powder particles are introduced into the gas stream upstream of the nozzle. The high-speed gas flow accelerates the particles to velocities typically between 300 and 1200 m/s [11,12]. When the high-velocity particles collide with the substrate, their kinetic energy is converted into plastic deformation energy. This energy causes the particles to undergo severe plastic deformation, which is critical for bonding. The impact results in the mechanical interlocking and metallurgical bonding of the particles to the substrate and to each other. The process forms a dense, adherent layer without significant heating, melting, or phase changes. The process is repeated layer by layer to build up the desired geometry. Each successive layer bonds to the previous one through the same high-velocity impact mechanism, resulting in a homogenous and dense structure.

### 2.2. Process Parameters

**Figure 4:**
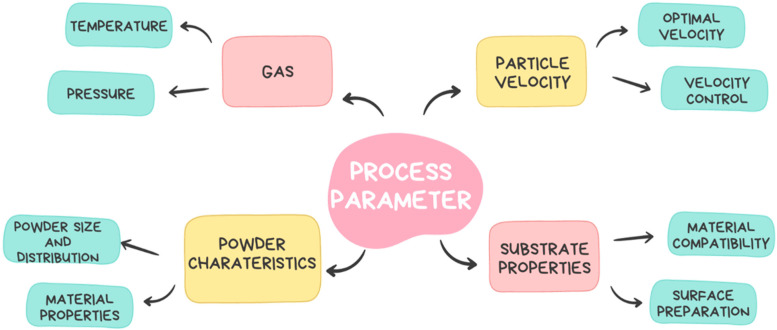
Process parameters.

Several critical parameters influence the quality, efficiency, and properties of the deposited material in CSAM. These parameters include gas temperature and pressure, particle velocity, powder characteristics, and substrate properties. These parameters influence the density, porosity, adhesion, and hardness of the printed parts. The process parameters for CSAM have been presented in Figure 4.

#### 2.2.1. Gas Temperature and Pressure 

Higher gas temperatures can increase the particle velocity by reducing the gas density and viscosity. However, the temperatures remain below the melting point of the particles, maintaining the solid-state nature of the process. Increased gas pressure enhances the acceleration of particles. Optimal gas pressure ensures sufficient particle velocities to achieve effective bonding without causing excessive wear on the nozzle or substrate. 

For instance, in a study by X. Meng, the effect of gas temperature on the properties of cold-sprayed coating was investigated. The results showed that increasing the gas temperature greatly strengthened the bonds between the deposited particles, producing a microstructure that was denser [13]. As a result, the coatings’ porosity dropped to 2% ± 0.3% from 6% ± 0.5%, and their tensile strength rose to 73 ± 3 MPa from 56 ± 4 MPa. Furthermore, the temperature of the process gas significantly affected the coatings’ ability to resist corrosion. The plastic deformation of the deposited particles and the decreased porosity in the coatings had an impact on the corrosion kinetics. Total coated thickness (TCT) strength increases noticeably from about 120 MPa to over 300 MPa when the temperature rises from 600 to 1000 °C. This corresponds to an ultimate tensile strength range of 180 MPa to 450 MPa, or bulk grade 3 titanium, when accounting for a notch factor of 1.5. There are two main aspects that are related to strength enhancement. First, the particles’ kinetic energy is increased by greater gas temperatures. Gas achieves sonic speeds near the throat of the nozzle; for ideal gasses, these speeds correspond exactly to the square root of the temperature. Ideal gasses are the ones which follow the ideal gas law and exhibit predictable behavior under different conditions of temperature and pressure. Higher impact velocities and more effective particle acceleration are the outcomes of this technique. Second, higher temperatures cause the spray material to become more ductile, which lowers the critical velocities required to produce shear instabilities.

#### 2.2.2. Powder Characteristics

The size and distribution of powder particles affect their acceleration and impact behavior. Smaller particles achieve higher velocities but may have lower mass, affecting their energy impact. A narrow size distribution ensures consistent deposition. The hardness, ductility, and density of the powder material influence its behavior during impact. Ductile materials are generally more suitable for cold spray, as they deform plastically and bond more readily. In CSAM, the properties of the powder are critical in determining the behavior of deposition and the quality of the resulting materials. Unlike traditional heat-based additive-manufacturing methods, CSAM operates as a solid-state deposition process, where powder particles are accelerated and deposited onto a substrate at high velocities without melting. This preservation of the powders’ inherent characteristics makes their properties essential to the final quality of the deposits [14].

One of the most significant powder properties in CSAM is the oxygen content. Powders with low oxygen levels tend to produce higher-quality deposits because there are fewer oxides that can interfere with the bonding process. High oxygen content can lead to brittle oxide films on the powder surfaces, which may break during high-speed impact and become trapped at the interfaces between deposited particles. This inclusion of oxides can prevent effective bonding and degrade the mechanical properties of the deposits. For instance, research has shown that reducing the oxygen content in copper powders from 0.12 wt.% to 0.044 wt.% can enhance the tensile strength from 197 MPa to 245 MPa and improve electrical conductivity from 85% IACS to 90% IACS [15]. The tensile strength test was carried out along the nozzle-traverse direction or the in-plane direction so that the anisotropy created due to the layer-by-layer deposition of the powder could be neglected. According to Chu et al. [16], lowering the oxygen percentage of Ta powder can encourage the powder particles to generate a higher flat rate and improve the mechanical interlock, which will increase the deposits’ tensile strength. This is because oxides on powder particle surfaces can decrease cold-spray-deposit-bonding characteristics [17,18,19,20,21]. The interface between the deposited particles contains some oxides, which inhibit the interface’s ability to form an effective binding [14,22,23].

Particle size and morphology also play vital roles in CSAM. The size of the powder particles influences deposition efficiency and the microstructure of the deposits. Smaller particles typically result in denser and finer microstructures, while larger particles can produce coarser deposits. Spherical particles offer better flowability and more uniform deposition compared to irregularly shaped particles, leading to smoother and more consistent deposit surfaces. Additionally, the internal microstructure of the powder, including grain size and phase composition, can significantly affect the properties of the final deposit, with refined microstructures generally enhancing the mechanical properties by providing uniform deformation characteristics during impact. However, maintaining the low oxygen content of powders poses challenges, as these powders are prone to oxidation during transportation and storage, often necessitating re-pulverization to retain their low-oxygen state. Production techniques like gas atomization or radio frequency plasma spheroidization are typically used to produce powders with the desired low oxygen content and characteristics, but these methods can increase the cost of the powders and, consequently, the overall cost of the CSAM process. By carefully optimizing the powder property-oxygen content, particle size, morphology, and microstructure, CSAM can achieve high-quality, dense, and mechanically robust deposits, making the selection and preparation of powders crucial for the success of the process.

#### 2.2.3. Particle Velocity

**Figure 5:**
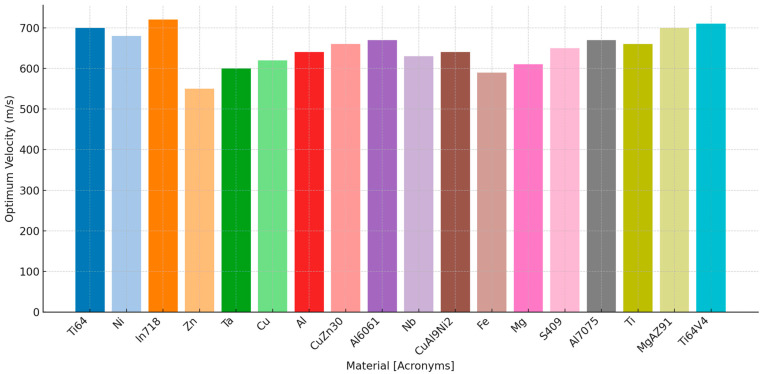
Deposition velocity range for cold spray across various materials [24].

There is a critical velocity for each material, below which particles may not adhere effectively, and above which particles may erode the substrate. Achieving optimal velocity is crucial for maximizing deposition efficiency and coating quality. Adjusting the gas flow rate, nozzle design, and stand-off distance (distance between nozzle and substrate) helps control the particle velocity. The velocity at which the particle is being sprayed must be within an optimal range, which is also known as the window of deposition for the cold-spray process. This window varies with the material being used. A summary of the deposition velocity range for cold spraying across various materials is presented in Figure 5.

#### 2.2.4. Substrate Properties

The substrate material must be compatible with the powder to ensure good bonding. Similar thermal expansion coefficients between the substrate and deposited material can reduce residual stresses. The substrate surface should be clean and, in some cases, roughened to enhance mechanical interlocking of the particles. Surface oxidation or contamination can impede bonding. Z. Arabgol verified the influence of substrate properties on the deposit properties [25]. In the study, the electrical conductivity of the coatings was assessed on the upper surface of coatings that were (0.8–1 mm) thick to gauge the quality of the coating. It was discovered that the substrate’s thermal effusivity and starting temperature had a significant impact on the coating conductivity. Only in areas that are 50 mm or less from the substrate/coating interface can the mechanical characteristics of the substrate additionally affect the local coating qualities. In another study, the effects of surface roughness on Ti6Al4V (Ti64) coatings deposited on Ti64 substrates were examined [26]. From 0.05 µm (polished) to 5.4 µm (waterjet cut), the surface roughness varied. The porosity, hardness, and coating surface roughness were found to be primarily impacted by deposition parameters such as propellant gas pressure, temperature, and nozzle traverse speed, and were not significantly affected by substrate roughness. Nevertheless, the coating bond strength was much increased by smoother substrates. It increased from roughly 7.1 MPa on rough surfaces (5.4 µm) to 68.7 MPa on smooth surfaces (0.05 µm). On smoother surfaces, the fracture characteristics showed more adiabatic shear-induced craters. Another study by A. Bruera examined the impact of substrate hardness and roughness on the adhesion of cold-sprayed copper onto AISI 304 stainless steel [27]. It found that roughened and hardened substrates reduced adhesion, while roughened and annealed (softened) substrates increased adhesion, peaking at a roughness (Rz) of about 34 µm. Grit-blasting work-hardened the surface, while vacuum annealing restored lower hardness without altering roughness. The adhesion differences were linked to particle deformation and substrate surface topographies, highlighting the importance of substrate properties in optimizing cold-sprayed coating adhesion.

#### 2.2.5. Nozzle Design

The design of the nozzle affects the gas flow dynamics and particle acceleration. A well-designed nozzle ensures uniform particle distribution and consistent deposition. The distance between the nozzle and substrate influences the particle velocity at the point of impact. An optimal stand-off distance balances particle acceleration and spread. The diameter of the nozzle in CSAM greatly affects the deposition efficiency and particle velocity. Denser deposits are produced when titanium alloy particle velocity is increased, as reported in the literature [28]. Laval nozzles with convergent and divergent portions must be properly built to achieve high particle velocity. Particle acceleration is significantly influenced by the interior nozzle dimensions, such as the divergent length and expansion ratio [29]. Most of the research focuses on divergent length and nozzle expansion ratio (exit to throat cross-sectional area) optimization [30,31,32,33]. The energy-efficient spraying capabilities of micronozzles with Al and Ti powders have been studied [34,35,36,37], and the effect of convergent length on Al powders has been examined [34]. Additionally, studies have focused on the impact behavior of single Ti particles during deposition procedures [38] and the creation of single-pass Ti coatings with various nozzle configurations [39]. The research highlights the significance of nozzle size in improving the accuracy and caliber of CSAM procedures.

**Figure 6:**
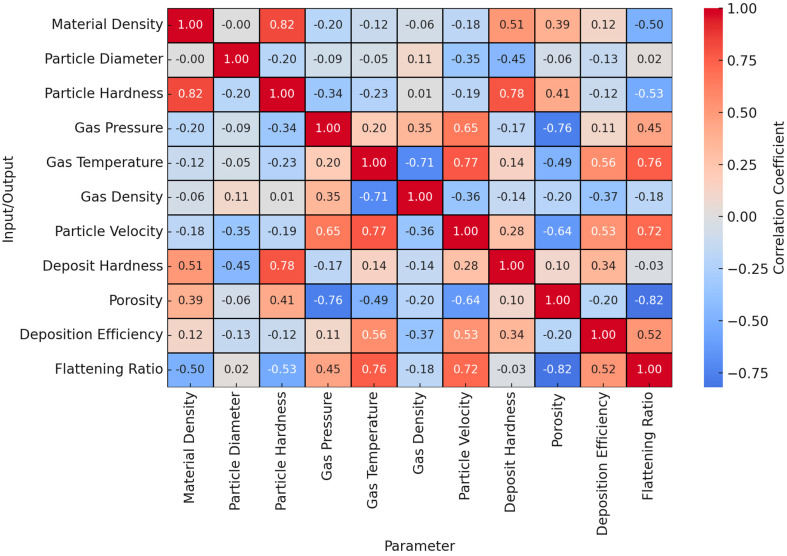
Correlation behavior of different input and output parameters [40].

Figure 6 illustrates the correlations between various process parameters and their effects on material density, particle diameter, particle hardness, gas pressure, gas temperature, gas density, particle velocity, deposit hardness, porosity, deposition efficiency, and flattening ratio. The correlation heatmap reveals important relationships between process parameters and material properties. Material density shows a strong positive correlation with particle hardness (0.82) and deposit hardness (0.51), suggesting that as the density increases, both particle and deposit hardness also increase. However, material density has a notable inverse relationship with the flattening ratio (−0.50), indicating that denser materials tend to experience less flattening upon impact. Particle diameter, on the other hand, has generally weak correlations with other parameters, with the strongest inverse relationship being with deposit hardness (−0.45), implying that larger particles may result in softer deposits. Particle hardness is positively correlated with deposit hardness (0.78), indicating that harder particles lead to harder deposits, while a moderate inverse relationship with flattening ratio (−0.53) suggests that harder particles flatten less upon impact. Gas pressure has a positive effect on particle velocity (0.65) and flattening ratio (0.45), indicating that higher gas pressure results in faster-moving particles and more flattening. Additionally, gas pressure is inversely correlated with porosity (−0.76), meaning that increased gas pressure reduces porosity, leading to a denser material. Gas temperature plays a significant role, showing strong positive correlations with flattening ratio (0.76) and particle velocity (0.77), suggesting that higher temperatures result in increased particle velocity and more particle spreading. In contrast, gas temperature has a strong inverse correlation with gas density (−0.71), indicating that an increase in temperature lowers gas density. Similarly, gas density shows a moderate negative relationship with particle velocity (−0.36), suggesting that higher gas density results in slower particles. Particle velocity is positively correlated with flattening ratio (0.72) and deposit hardness (0.28), indicating that faster-moving particles lead to more flattening and harder deposits. Additionally, particle velocity has an inverse relationship with porosity (−0.64), suggesting that higher velocities reduce porosity. Deposit hardness is strongly related to particle hardness (0.78) and has a slight inverse relationship with porosity (−0.10), implying that harder deposits tend to have lower porosity. Porosity itself is inversely correlated with both flattening ratio (−0.82) and gas pressure (−0.76), meaning that as particle flattening or gas pressure increases, porosity decreases, resulting in denser deposits. Deposition efficiency is moderately correlated with both flattening ratio (0.52) and particle velocity (0.53), indicating that increased particle flattening and velocity improves how well particles adhere to the surface. The flattening ratio also has a strong inverse correlation with porosity (−0.82), suggesting that as particles flatten more, the material becomes less porous and more compact. In summary, the analysis highlights how optimizing process parameters, such as gas pressure, gas temperature, and particle velocity, can lead to denser, harder deposits with improved deposition efficiency and reduced porosity, all of which are critical for enhancing material properties in manufacturing processes.

Table 1 presents the effects of several manufacturing settings on important deposit characteristics, including adhesion, residual stress, porosity, deposit strength, and deposition efficiency. In the first two rows, porosity is decreased while deposit strength, adhesion, residual stress, and deposition efficiency are increased with increasing gas temperature and pressure. This implies that stronger, denser, and more effective deposits are created at greater temperatures and pressures of gas. On the other hand, increasing the gas molecular weight results in larger porosity and worse deposition efficiency, adhesion, residual stress, and deposit strength. This suggests that the overall quality of the deposited material is adversely affected by heavier gases. Porosity rises with increasing powder feed rate, although deposition efficiency is improved. It does, however, diminish residual tension, adhesion, and deposit strength, suggesting that even though more material is deposited, its structural integrity is lost. On the other hand, as transverse speed increases, porosity decreases and deposit strength, adhesion, and efficiency are all improved; residual stress is also increased. This suggests that deposits become denser and stronger at greater speeds. Ultimately, a broader spray angle results in less deposit strength, adhesion, residual stress, and efficiency, as well as more porosity. This shows that for compact, powerful, and effective deposits, a smaller spray angle is preferable.

## 3. Materials

Cold spray additive manufacturing utilizes a high-velocity deposit of powdered materials to build solid layers without melting them. This technique primarily employs metals and metal alloys due to their unique properties that suit the cold spray process. A wide range of plastics, metals, and ceramics can be manufactured using this process [41]. Materials with a low melting point and low mechanical strength, such as zinc (Zn), aluminum (Al), and copper (Cu), are considered favorable due to their low yield strength and notable softening at high temperatures. Common materials include aluminum and its alloys, known for their lightweight and corrosion resistance, making them ideal for aerospace and automotive applications [42,43]. Copper and its alloys are used for their excellent thermal and electrical conductivity, while titanium alloys provide high strength-to-weight ratios and corrosion resistance, essential in aerospace and medical fields [44,45]. Nickel and stainless steel are favored for their durability and resistance to high temperatures and corrosion, suitable for industries like marine, medical, and energy [46,47]. Nickel based alloys have been used for restoration and surface enhancement on cast iron [48].

**Figure 7:**
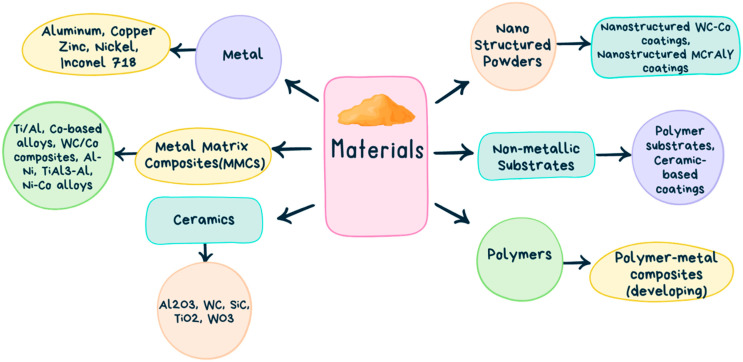
Classification of different commonly used material for the cold spray process.

**Figure 8:**
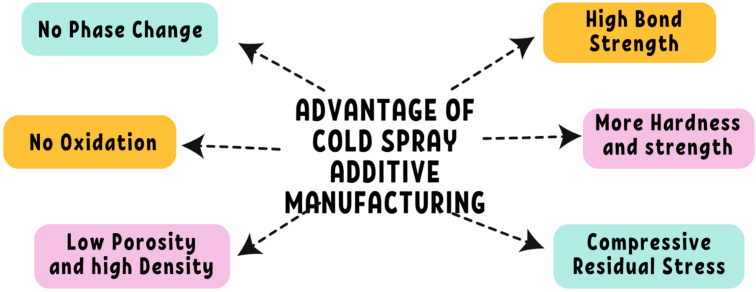
Various advantages of material processing with cold spray additive manufacturing.

Figure 7 provides a detailed categorization of various materials used in advanced manufacturing, particularly for coatings, composites, and substrates. Fundamentally, the materials can be divided into metals, ceramics, polymers, metal matrix composites (MMCs), non-metallic substrates, and nano-structured powders. Each of these groups offers special qualities that are appropriate for a variety of industrial uses. Because of their strength, resistance to corrosion, and ability to withstand high temperatures, metals like Inconel 718, aluminum, copper, zinc, and nickel are highly desired in the aerospace, automotive, and high-temperature industries. Metal matrix composites (MMCs)—such as Ti/Al, Co-based alloys, and WC/Co composites—are perfect for harsh environments because they blend metals with ceramics or other reinforcements to improve strength and wear resistance. Ceramics like Al_2_O_3_, WC, SiC, TiO_2_, and WO_3_ are good choices for cutting tools, wear-resistant parts, and high-temperature applications because of their remarkable hardness, thermal stability, and chemical resistance. Nano-structured powders, such WC-Co and MCrALY coatings, augment hardness and corrosion resistance at the nanoscale, hence augmenting the efficacy of coatings employed in difficult circumstances. Polymers and emerging polymer–metal composites are lightweight and flexible, offering promise for future applications. Non-metallic substrates, such as coatings based on polymers and ceramics, act as fundamental materials that improve strength and durability when covered. The combination of these material categories offers a wide range of high-performance possibilities that are utilized in contemporary industries, such as manufacturing and aerospace. Innovations in nano-structured and composite materials are primarily driven by ongoing research. Hard materials like tungsten and tungsten carbide are often used for wear-resistant coatings, while softer metals such as zinc serve corrosion protection. Although less common, precious metals like silver and gold are employed for specialized applications due to their conductivity and resistance to oxidation. Cold spray technology is advantageous for materials that are challenging to weld or melt, preserving their properties, and offering robust adhesion with minimal oxidation. Figure 8 lists some benefits of using CSAM.

### Material Selection Strategy

In CSAM, the choice of material dictates the success and performance of the final component. The journey begins with understanding the inherent properties of potential materials. Ductility and hardness are at the forefront; ductile materials such as copper, aluminum, and titanium stand out for their ability to deform plastically under high velocity impact, forging strong bonds with the substrate. On the other hand, harder materials like tungsten or ceramics demand a delicate balance of process parameters to avoid substrate erosion while ensuring robust adhesion. The melting point is equally crucial, as CSAM operates in a solid-state regime. Materials with lower melting points are prime candidates, preserving their intrinsic characteristics throughout the process and avoiding the phase transformations typical in high-temperature manufacturing methods. The powder characteristics further refine the selection process. The oxygen content in the powder is a vital factor—low-oxygen powders are the gold standard, ensuring minimal oxide formation during deposition. A high oxygen level can introduce brittleness, disrupting the integrity of the deposit. Therefore, selecting powders with a meticulously controlled oxygen content is essential to achieving seamless interparticle bonding and enhancing the overall performance of the component. Additionally, the internal microstructure of the powder, including grain size and phase composition, plays a defining role. Fine-grained powders are preferred for producing denser and more uniform deposits, which translate into superior mechanical properties such as increased tensile strength and hardness. Compatibility with the cold spray process is non-negotiable. Each material has a critical velocity threshold—a sweet spot where particles adhere effectively without eroding the substrate.

**Figure 9:**
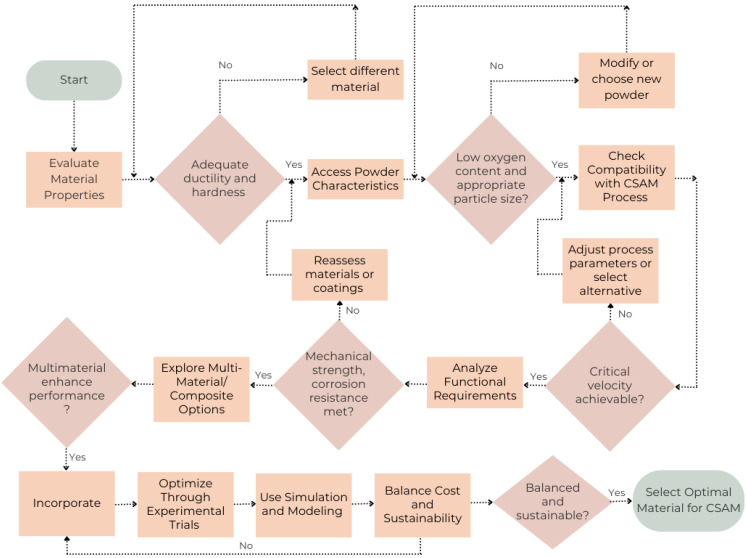
Material selection strategy for CSAM.

Achieving this balance is key to maximizing deposition efficiency and coating quality. Beyond critical velocity, thermal and mechanical compatibility must also be considered. The material’s thermal expansion coefficient should align closely with that of the substrate to minimize residual stress, ensuring that the deposit remains free of cracks and delamination. This careful pairing of substrate and powder material is a strategic move, setting the stage for a successful deposition process that results in components with reliable mechanical performance. The application-specific requirements drive the final choice of materials. For aerospace and automotive industries, the material must offer high mechanical strength and wear resistance. Titanium alloys, stainless steel, and nickel-based superalloys are the frontrunners in these scenarios, boasting excellent properties that endure under stress. In contrast, environments exposed to corrosive elements, such as marine or biomedical fields, demand materials like aluminum alloys, copper, and titanium, renowned for their corrosion resistance. For applications requiring optimal thermal and electrical conductivity, copper and silver are the materials of choice, providing the high conductive properties necessary for components like heat exchangers and electrical contacts. Embracing innovation, CSAM opens the door to multi-material and composite deposits. This strategy allows for the creation of functionally graded materials (FGMs), where properties transition smoothly across a single component. By selecting and blending different powders and fine-tuning process parameters, CSAM can fabricate parts with varying hardness or thermal conductivity, tailored to specific operational requirements. Composite coatings introduce another layer of versatility; by integrating ceramic particles or hard phases into a ductile metal matrix, the resulting deposits can exhibit enhanced wear resistance, hardness, and thermal stability. This approach allows for the design of materials with precisely engineered functional attributes, expanding the potential applications of CSAM. Material selection in CSAM is not a one-size-fits-all solution but rather an iterative process involving rigorous experimentation and optimization. Initial trials with various materials help determine their suitability, requiring adjustments in gas temperature, pressure, and stand-off distance to achieve optimal deposition characteristics. In parallel, simulation and modeling come into play, leveraging computational models and machine-learning techniques to predict material behavior during the cold spray process. These advanced tools allow for the evaluation of factors such as particle velocity, deformation, and bonding quality, streamlining the material selection process and reducing the reliance on trial and error. The basic overview of material selection strategy is discussed in Figure 9.

## 4. Enhancing Strength and Precision in Cold Spray Manufacturing: Advanced Strengthening Techniques, Computational Modeling, and Machine Learning

### 4.1. High-Strength Manufacturing

**Figure 10:**
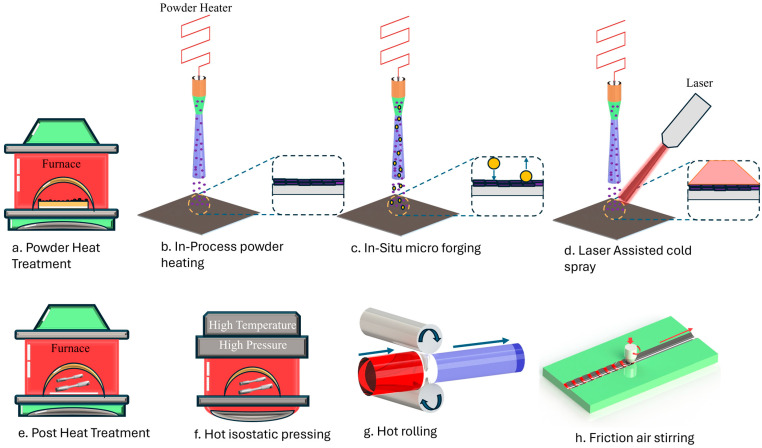
Available strengthening technologies for cold-spray deposits (Redrawn from [8]).

Cold-spray technology is particularly effective for producing coatings and components with enhanced mechanical properties [8]. The process avoids high temperatures that can alter material properties, making it ideal for sensitive materials. Studies have focused on optimizing the process parameters to achieve desired mechanical properties, such as hardness and wear resistance. For instance, a study on laser-heat-treated copper coatings demonstrated that optimizing laser parameters significantly improved hardness and reduced residual stress. A lot of different strengthening methods can be applied for strengthening the mechanical properties of the cold spray deposits. Based on their stage of application, strengthening technologies used in the cold-spray process can be divided into three groups, i.e., pre-process, in-process, and post-process. Various available strengthening techniques for cold-spray techniques have been illustrated in Figure 10.

Cold spraying started with the use of pre-process technology. For instance, powder heat treatment can enhance the quality of cold spray deposition by heating the powder to improve its qualities, such as reducing its hardness or increasing its ductility [49]. To enhance the coating quality and deposition properties, in-process technologies are implemented during the cold-spray process itself. This includes methods like in situ micro-forging, which mechanically deforms the particles during the process to improve bonding and coating density, and powder heating, which increases the plasticity and adhesion of powder particles upon impact. Another method is laser-assisted cold spray, which uses a laser to heat the substrate or powder particles locally to improve coating properties and deposition efficiency [50,51,52,53]. Compared to traditionally cold-sprayed deposits, LACS deposits exhibit higher deposition efficiency, and the deposition efficiency rises as laser power increases. Increased interparticle metallurgical bonding results from enhanced plastic deformation and high-temperature-induced atomic diffusion between interparticle contacts [54]. In-process powder heating is another strengthening mechanism. Powder heating during the procedure has led to a notable increase in the densities of metallic glass deposits [55], Ni-coated FeSiAl composite [56], Cu [57], and Ni [58,59]. Post-process strengthening can also be employed to improve the strength of the CSAM parts. For cold-sprayed deposits, the most popular, practical, and affordable strengthening method is conventional post-spray heat treatment [1]. The microstructure of cold-sprayed Ti6Al4V deposits was analyzed in both their as-fabricated condition and after undergoing annealing at various temperatures. The fraction of high-angle grain boundaries (HAGBs) increases significantly in the annealed deposit, but the fraction of low-angle grain boundaries (LAGBs) decreases, suggesting that recrystallization has occurred. It is also critical to emphasize that the grain size increases with increasing annealing temperature [60]. Hot rolling has also been used as a mechanical post-processing technique for deposits that were cold sprayed. To apply strong plastic deformation, the cold-sprayed deposits are heated in a furnace to a temperature higher than the recrystallization point and then run through a rolling device [61,62,63,64]. Friction stir processing (FSP) has also been adapted as a post-processing technique for enhancing the strength of cold-sprayed deposits [65,66,67]. The grain structure of the FSP-treated deposit becomes homogenous because of recrystallization brought on by the elevated processing temperature. This suggests that while refining the grain structure and effectively eliminating flaws like pores and weak interparticle boundaries, FSP can also reinforce the material. 

### 4.2. Computational Modeling

To optimize the cold-spray process, sophisticated computer frameworks and simulation tools are being developed. These methods minimize the need for lengthy experimental runs by optimizing process parameters and predicting deposition outcomes using mathematical models and machine-learning techniques. Convolution techniques and Gaussian approximations, for instance, have been applied to optimize spray parameters and forecast deposition forms. Digital simulation can be employed for three-dimensional modelling of cold spray additive manufacturing. For example, S. Garmeh investigated the application of CSAM simulation in three dimensions for a high-pressure nozzle using axial powder injection [10]. The study investigates how stagnation spots, corners, sharp edges, and sloped surfaces affect spraying precision and particle trajectories in CSAM. Stronger bow shocks near cylinders caused particle dispersion and frustum-like deposition patterns, according to simulations employing a cylinder and frustums at varying angles. Compared to the 45° frustum, the 15° frustum had less side deposition. Particle dispersion limited the deposition precision to about 6 mm. Small-diameter injectors, non-circular nozzles, and tilting the nozzle are advised to increase precision; more simulations are suggested for further optimization.

**Figure 11:**
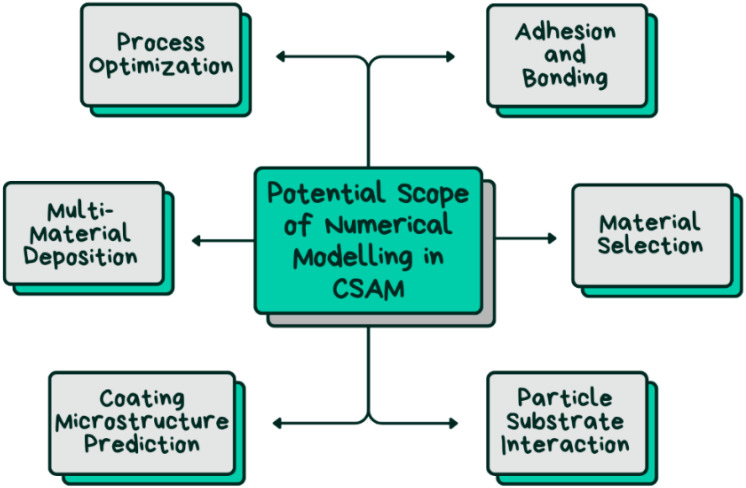
Future of cold-spray-additive-manufacturing-modeling process.

Daniele et al. reported the development of a 3D model with the ability to simulate the cold spray deposit profile [68]. The model depicts the evolution of the deposit profile with an increasing number of passes using a partial differential equation. Complete control over the nozzle trajectory and substrate geometry is granted, and the implications of important process variables like the number of scanning passes, spray angle, scanning speed, and stand-off distance are considered. It can also replicate intricate situations like non-Gaussian profiles, curved substrates, shadow effects, and superimposed tracks with different spray angles. Extensive experiments have been conducted to validate the model, which has demonstrated great accuracy and significant potential to improve the digitalization of CSAM. Additionally, in [69], a computationally efficient 3D deposition model for CSAM has been developed, which uses a gradient-descent-based tool-path optimization algorithm to adjust velocity and achieve precise convex deposit shapes. The model, calculated through experiments, generates optimized robotic g-code for a 6-axis arm based on input STL files and is validated by applying deposits on convex surfaces. The potential scope of future cold-spray-modeling processes is presented in Figure 11.

S. Garmeh et al. simulated CSAM for shapes like cylinders and frustums, examining particle trajectories and impact conditions to address deposition challenges in complex geometries [70]. The modeling results validated against experiments showed accurate predictions of shock diamonds and bow shocks. For a flat substrate, a strong bow shock forms, influencing particle velocities. When manufacturing on a cylinder, the strong bow shock at the top repels higher-velocity particles to the edges, reducing deposition precision. In the frustum case, weaker shocks allow more particles to hit the sides at lower velocities, risking weak bonding. High-velocity particles are redirected to the base, possibly smearing the frustum’s slope, challenging vertical structure formation and deposition accuracy.

**Figure 12:**
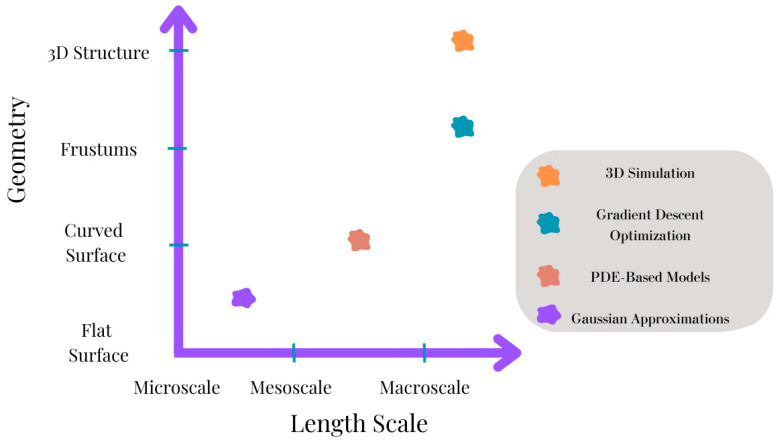
Geometry vs. length scale for cold-spray-modeling approaches.

Figure 12 presents a comparative analysis of various cold-spray-modeling approaches based on their geometry and length scale. This type of figure typically categorizes and maps different modeling methods, ranging from micro-scale to macro-scale, to correspond to specific geometrical complexities.

### 4.3. Machine-Learning Integration

**Figure 13:**
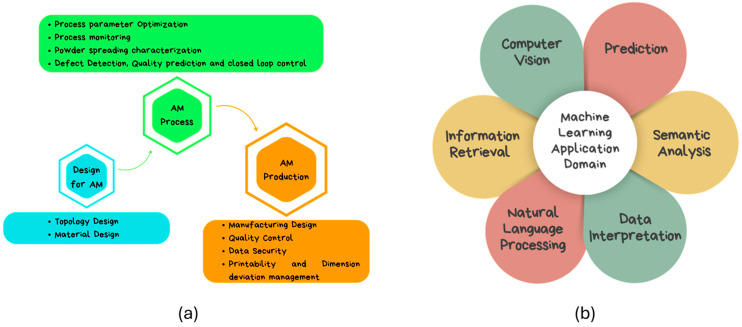
(**a**) ML approaches applied in several AM domains; (**b**) key areas of use for ML (redrawn from [79]).

Machine learning (ML) has become more and more common in manufacturing in recent years, saving labor costs, time, and effort. Product quality has increased, while time, energy, and resource savings have been realized through the integration of machine learning (ML) with sophisticated digital manufacturing procedures and data availability. Through the automatic, real-time correction of faults to minimize waste, machine learning (ML) in manufacturing encourages intelligent operations. Machine-learning models are increasingly being integrated into cold spray processes to predict and control various aspects of the deposition. Artificial neural networks (ANNs), support vector machines (SVMs), and other algorithms have been employed to predict critical process parameters, such as porosity, adhesion, and hardness [71,72,73,74]. These models have shown high accuracy and reliability in predicting outcomes based on input parameters. For instance, deposition efficiency can be predicted using both regression (first-order polynomial and second-order polynomial) and classification models (kNN, logistic regression, random forest, and SVM) with accuracy up to 0.98 and 0.99 [75]. In [75], machine learning has also been utilized to predict deposition efficiency in cold spraying by identifying an energy threshold for particle adherence. The models provide insights into the cold-spraying process, enabling accurate predictions for various powder/substrate systems. A data-driven, overlapping track–profile can also be modeled in CSAM. D. Ikeuchi modeled a surface-aware Gaussian process regression model that incorporates domain knowledge, resulting in improved predictions and enhanced product quality [76]. Cold spray input parameters can also be predicted using CSAM. In [77], a machine-learning-based inverse-modeling technique is proposed to predict input process parameters (IPPs) for achieving desired SDPs, demonstrating accuracy and reliability in simulations. Optimization algorithms are used to fine-tune the cold spray process parameters, leading to improved efficiency and reduced material waste. Machine-learning-based optimization frameworks can identify the best combinations of parameters to achieve desired properties, thus enhancing the overall performance of the cold-spray process. In [78], spray trajectory planning was computed for complex structural components in a robotized CSAM. Figure 13 highlights different ML approaches applied in AM domain and shows different key areas of application of ML [79].

Generative models, such as generative adversarial networks (GANs), are used to create realistic simulations of cold-spray processes. These models can generate synthetic data for training machine-learning algorithms, enabling better prediction and control of the process without extensive experimental data. This approach is particularly useful for optimizing complex processes like CSAM. In [80], a novel approach to model complex microstructures in cold-spray-formed (CSF) aluminum alloys by integrating generative adversarial networks with synthetic microstructure builders. The method generates statistically equivalent virtual microstructures (SEVMs) for accurate finite element simulations, capturing the material’s morphological and crystallographic features. 

## 5. Innovative Applications of Cold Spray Additive Manufacturing: Structural, Thermal, and Repair Solutions

Many different components, such as tubes, flanges, and cylinder walls, have rotating shapes. A straightforward spray technique makes it easy to create the rotating structure by using an external spindle to support the mandrel substrate. CSAM can also be used to create the outside and inner walls of metal cylinders [81,82,83,84,85]. Particularly in the case of an outside wall, the fabrication procedure is comparable to that of flange manufacturing. An example of a 1/10 sized canister made with CSAM for the disposal of CANada Deuterium Uranium (CANDU) spent fuels [82]. Good mechanical stability, thermal stability, and tensile strength were achieved when a 10 mm thick layer of copper with a porosity of 0.3%, density of 8900 kg/m^3^, and oxygen content of 0.019% (as opposed to 0.02% in the feedstock copper powder) was placed onto the cast iron cylinder. Complex structures can also be produced using cold spray manufacturing in conjunction with subtractive manufacturing. For example, M.E. Lynch and colleagues demonstrated how to manufacture intricate structures with cold spray technology. These structures were created with advanced structural optimization in mind; 60% less stress and 20% less weight was achieved by optimizing the geometry of the structure [86]. Furthermore, CSAM can also be used to repair damaged parts. In the past, CSAM has been used by many for repairing damaged components. For complex structures, cold spraying can be directly executed on a complex surface topology for repairing parts [87,88].

**Figure 14:**
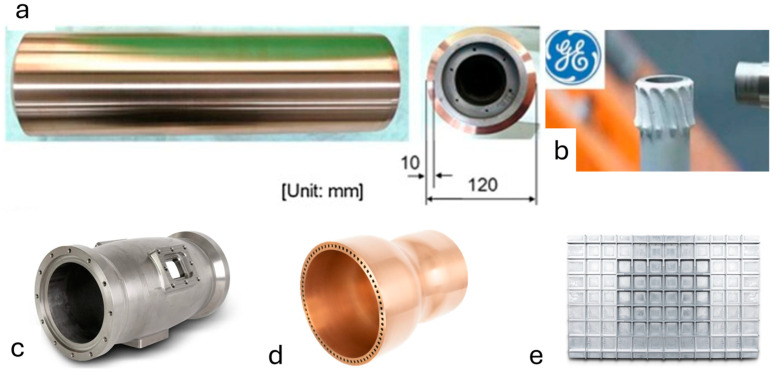
Manufacturing different structures using CSAM: (**a**) scaled canister for disposal of CANDU spent fuels [82]; (**b**) Gear [1]; (**c**) rotational symmetrical casing [3]; (**d**) combustion chamber [3]; (**e**) ortho-grid structure [3].

Array structures can also be produced using CSAM. A carefully made mask is required for the fabrication process to limit the number of superfluous materials deposited and only permit the desired pattern to be deposited. The pyramidal fin arrays for a compact heat exchanger, as seen in Figure 14 [89,90,91,92,93], are an illustration of a CSAM array structure. O. Tregenza used CSAM for manufacturing thermal electric generator thermal interfacing [94]. This research explores the impact of thermal interface resistance on thermoelectric generator (TEG) performance, showing that high clamp forces and fine surface finishes minimize resistance and power loss. Additionally, cold spray copper interfaces further reduce resistance, leading to minimal power loss. Furthermore, P. Kindermann proposed cold spray forming as a novel approach in CSAM of complex parts using 3D-printed polymer molds [95]. The paper demonstrates that molds produced via rapid tooling with MEX-TRB/P for cold spray forming (CSF) offer a novel approach to improving the process, though material deposition was only successful with PEEK due to its sufficient hardness at elevated temperatures. Key findings include the significant impact of print quality on sprayed layers, geometric limitations related to transition angles, and the continuation of surface textures into sprayed layers. Future advancements require refining the geometric limits, validating mold dissolution methods, and improving the quality of PEEK parts.

Cold spray technology was employed to restore the dimensions and functionality of the A357 cast aluminum alloy component in the F/A-18E/F Super Hornet fighter [96]. Moreover, the Army Research Laboratory, in collaboration with General Electric and Moog, repaired the GE T700 Front Frame Housing using cold-spray technology. The front frame, originally made from cast C355 aluminum alloy, was repaired with 6061 aluminum alloy [97]. Additionally, cold spray was utilized to repair components of Seahawk helicopters, offering a cost savings of 35–50% compared to manufacturing new parts [96]. In another study, pure copper with outstanding strength and ductility was successfully produced using CSAM, marking a significant breakthrough in the field [98].

## 6. Overcoming Challenges in Cold Spray Additive Manufacturing: Material, Process, and Environmental Hurdles

The widespread acceptance and deployment of CSAM is impeded by multiple significant difficulties [99,100]. Ensuring strong adhesion and bonding between the deposited material and the substrate is one of the main problems [101]. Since materials are deposited through high-velocity particle impact rather than melting, the bond in CSAM is essentially mechanical. This can result in weak bonding, which makes it challenging to guarantee consistent adhesion throughout a substrate, particularly when working with materials that range significantly in surface energy or mechanical properties [102]. Compatibility of the substrate is another significant challenge because not all substrates react favorably to the cold spray method. Certain substrates might cause damage during deposition or poor bonding because they are either too soft or too rigid. Furthermore, the success of bonding is strongly impacted by certain surface characteristics, such as cleanliness or roughness, necessitating meticulous control over substrate preparation.

The difficulty of accomplishing multi-material depositions in CSAM is another challenge. To create functionally graded materials, it is advantageous that the technique permits the simultaneous deposition of many components [103]. However, non-uniform depositions can emerge from variances in material qualities, such as hardness, density, and melting temperatures, which can cause weak spots in the finished product. The considerable energy needed to use pressurized gas to accelerate particles to supersonic speeds is a hurdle to the process efficiency of CSAM. This increases the energy intensity of the process, and inefficiencies in material waste or particle impact can raise operating costs and lower overall process efficiency. Furthermore, within CSAM, material development is still a developing field. Cold spray processing is not suitable for many materials, including those that frequently need significant alterations or customizations to make them process-optimized, for example, by having particle sizes and shapes.

The performance of the original specimen after cold-spraying repair may not always be fully restored, as it depends on several key factors, including material properties, repair techniques, and process parameters. Cold spray is an effective method for restoring structural integrity without introducing significant thermal stresses or material degradation, but there are limitations that can affect the overall performance of the repaired component. One major limitation is that the mechanical properties of the repaired area, such as tensile strength, fatigue resistance, and hardness, may not completely match those of the original material, especially in high-stress applications. The surface finish and precision of cold spray repairs can also be an issue, often requiring additional post-processing such as machining or polishing to meet the original specifications, particularly for components with complex geometries or tight tolerances. Another limitation is the potential for material mismatch between the sprayed material and the substrate, which can affect performance in terms of thermal expansion, corrosion resistance, or electrical conductivity. Cold-sprayed deposits may exhibit porosity, and the microstructure of the deposited material might differ from that of the original, which can negatively impact properties like ductility or impact resistance. The bonding strength between the cold-sprayed material and the substrate, while generally strong, may not always be as robust as the original material, particularly in components subject to high loads or complex shapes. Additionally, although cold spray avoids the thermal stresses associated with other repair methods, residual stresses can still develop from the high-velocity impact of particles, potentially leading to long-term issues like cracking or delamination in cyclic or high-stress environments.

Surface preparation is a crucial component of CSAM and is necessary to guarantee adequate adhesion. To maximize bonding, the surface needs to be thoroughly cleansed, roughened, and perhaps even pre-treated. Poor adherence or flaws in the coating, such as delamination or porosity, can be caused by inadequate surface preparation.

Another technological problem is controlling the thickness of the coating. CSAM is frequently used for thin, homogeneous coatings; however, because of factors, including nozzle distance, particle velocity, and powder feed rate, it can be challenging to maintain a constant thickness over complex geometries. The final product’s durability and performance may be impacted by this discrepancy. Production becomes more challenging when these parameters are changed without compromising other process elements like adhesion or material integrity.

**Figure 15:**
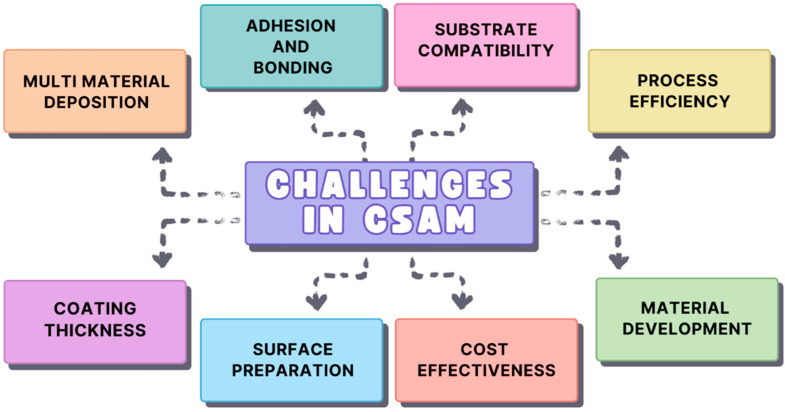
Challenges in cold spray additive manufacturing.

Lastly, there is growing concern about the environmental impact and sustainability of CSAM procedures [104]. The overall environmental impact is influenced by the demand for pressurized gas, the creation of metal powders, and the energy needed to accelerate particles. Reducing resource intensity and increasing energy efficiency will be essential for CSAM to stay competitive as industries shift to greener technologies [105]. Manufacturers of cold spray are actively working to come up with creative ways to lessen the negative environmental effects that come with using this technology [105,106]. In conclusion, even though CSAM has a lot of potential for temperature-sensitive, high-quality material deposition, overcoming these obstacles will necessitate continuous advancements in material science, equipment development, and process optimization to realize its full potential across industries [107]. The overall challenges in CSAM is presented in Figure 15.

## 7. Conclusions

CSAM is a significant advancement in additive manufacturing, offering unique capabilities across multiple industries. Unlike traditional thermal processes, CSAM operates at lower temperatures, minimizing thermal distortion and preserving the intrinsic properties of feedstock materials. This allows us to produce parts with enhanced mechanical strength and durability, essential for high-demand sectors like aerospace, defense, and automotive industries. The integration of computational tools and machine learning (ML) has been a breakthrough in optimizing CSAM. Real-time data analytics allow manufacturers to fine-tune parameters such as particle velocity, gas flow, and substrate temperature, improving deposition efficiency while reducing material waste and post-processing. ML models can predict optimal conditions, addressing issues like poor adhesion and porosity that were common in early CSAM iterations. The versatility of CSAM in handling a broad range of materials, including metals, polymers, and composites, makes it highly adaptable for producing complex geometries and functionally graded materials, often difficult to achieve with conventional methods. It also enables the coating, repair, and rebuilding of components with minimal heat-induced changes, promoting economical and environmentally friendly manufacturing.

## 8. Future Work

Despite progress, several challenges hinder the widespread industrial adoption of CSAM. Control over process parameters, such as cooling systems, particle acceleration, and nozzle design, remains a difficulty. While advancements in AI and simulations have helped streamline production, further improvements are needed to achieve industrial scalability. Looking ahead, CSAM’s potential spans beyond its current applications, with promising opportunities in space exploration, medical device production, and energy sectors. Its ability to produce or repair parts using high-performance materials positions it as a leading tool in modern manufacturing. However, sustained research in material development, process optimization, and digital integration is necessary to overcome existing barriers. The training framework discussed in [108] is a good reference for increasing the uptake of additive-manufacturing processes in the industry. Once these challenges are addressed, CSAM could become a key technology in advanced manufacturing, offering high-performance and eco-friendly solutions across industries.

## Figures and Tables

**Table 1:** Effect of manufacturing parameters on deposit properties [1].

	Porosity	Deposit Strength	Adhesion	Residual Stress	Deposition Efficiency
Gas Pressure	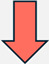	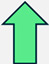	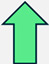	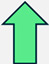	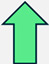
Gas Temperature	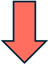	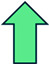	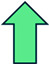	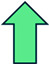	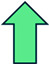
Gas Molecular Weight	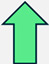	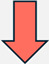	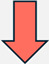	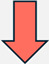	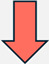
Powder Feed Rate	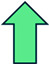	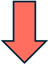	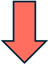	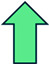	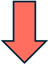
Transverse Speed	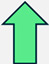	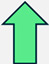	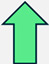	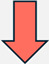	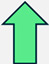
Spray Angle	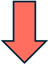	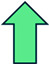	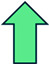	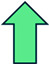	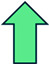

## Data Availability

No new data were created or analyzed in this study.
